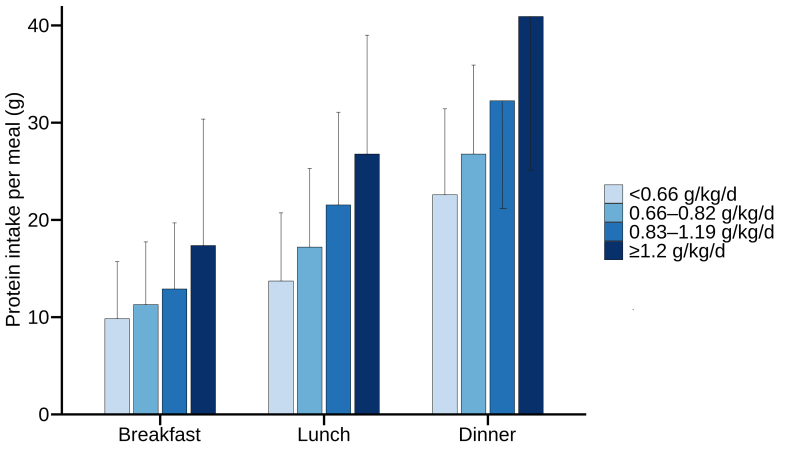# Differences in Protein Quantity and Quality Across a Spectrum of Plant-Based Meals: Analysis of a Large National Dietary Survey

**DOI:** 10.1016/j.cdnut.2026.107641

**Published:** 2026-01-22

**Authors:** Sophie L van Oppenraaij, Sjors Verlaan, Peter JM Weijs

**Affiliations:** 1 Department of Nutrition and Dietetics, Faculty of Health, Sport and Physical Activity, Amsterdam University of Applied Sciences, Amsterdam, The Netherlands; 2 Amsterdam Public Health, Aging and Later Life, Vrije Universiteit, Amsterdam, The Netherlands; 3 Department of Surgery, Radboud University Medical Center, Nijmegen, The Netherlands; 4 Department of Nutrition and Dietetics, Amsterdam University Medical Centre, Vrije Universiteit, The Netherlands

**Keywords:** animal-based, dietary intake, plant-based, protein intake, protein quality, protein transition, sustainable diets

## Abstract

**Background:**

Current recommendations encourage consuming sufficient intake of high-quality protein, with ≥60% derived from plant-based sources, to support both nutritional requirements and sustainability goals.

**Objectives:**

This observational study assessed protein intake, quality, and sources in predominantly plant-based meals and diets using a national survey, offering insights to support a more sustainable and nutritionally adequate dietary transition.

**Methods:**

In the Dutch National Food Consumption Survey (2019–2021), protein intake was assessed using 24-h recalls in adults aged 18 to 79 y. Total, plant-based, and animal-based protein intake were analyzed per meal and per day. Protein quality per meal was evaluated using the Meal Protein Quality Score (MPQS). The association between protein quantity and the proportion of plant-based protein was evaluated based on how often meals reached adequate protein quality (MPQS >100). Furthermore, protein sources across food groups in diets with ≥60% and <60% plant-based protein were compared.

**Results:**

Among 1747 adults [57 (44–68) y, 50% male], the median protein intake was 0.93 (0.75–1.13) g/kg/d. Only 8% (*n* = 147) had a diet comprising ≥60% plant-based protein, with a median intake of 0.83 (0.63–1.05) g/kg/d. As the proportion of plant-based protein increased, both protein quantity and quality decreased. When protein quality was low, lysine was the most common limiting amino acid. Only 3% of all meals achieved ≥20 g protein, ≥60% plant-based protein, and optimal protein quality, with dairy as key protein source at breakfast and lunch, meat alternatives at dinner, and grains at all meals.

**Conclusions:**

This study shows that only a small proportion of Dutch adults met both protein-related recommendations and sustainability goals, due to lower protein quantity and quality in more plant-based diets. This study emphasizes the need for professional guidance, especially in individuals with higher protein requirements, to facilitate a successful transition to a more plant-based diet.

## Introduction

Adequate protein intake is essential to maintain muscle mass and function across adulthood [1,2]. Loss of muscle mass, strength, and function, particularly with advancing age, can contribute to sarcopenia and is associated with higher chances of physical disability, falls, fractures, mortality, and with a lower quality of life. At the same time, dietary shifts toward more plant-based foods are promoted to improve cardiovascular health and reduce environmental impacts [[3], [4], [5], [6], [7]]. However, increasing the proportion of plant-based foods in the diet may affect both protein quantity and protein quality, underscoring the need to evaluate protein adequacy within more plant-forward dietary patterns [6,[8], [9], [10]].

The estimated average requirement (EAR) for adults is 0.66 g protein per kg body weight per day (g/kg/d), whereas the recommended dietary allowance (RDA) to meet the needs of most adults is 0.83 g/kg/d [[11], [12], [13]]. To maintain or (re)gain muscle mass, resistance exercise is recommended in combination with a protein intake of ≥1.2 g high-quality protein/kg/d, particularly in older adults [2,[14], [15], [16]]. Muscle protein synthesis has been shown to be optimally stimulated by 20 g or 0.24 g/kg of high-quality protein per meal in young adults [17,18], whereas older adults require a higher amount, typically 25 to 30 g or 0.4 g/kg per meal [[19], [20], [21]]. However, in practice, even 20 g of protein per meal is often difficult to achieve, particularly at breakfast [22,23].

Sufficient protein intake is usually realized by increasing intake of animal-based food products or supplements because those products have a high protein content and protein quality [[24], [25], [26], [27]]. However, increasing the proportion of plant-based foods in the diet is generally associated with a reduction in total protein intake [28,29], and protein from many plant sources (e.g., soy, legumes and grains) tends to have lower quality, digestibility, and absorption compared to animal-based foods [6,[8], [9], [10]]. Accordingly, lower acute anabolic responses have been observed following plant-based compared with animal-based protein ingestion in some [[30], [31], [32], [33]], but not all [34,35]. At the same time, animal-based food production contributes substantially to global greenhouse gas emissions, and high meat consumption has been linked to an increased risk of certain diseases [6,36]. Partly replacing animal-based food products with plant-based food, such as legumes, nuts, and vegetables, is a way to lower the environmental footprint and the risk of noncommunicable diseases (i.e., cardiovascular diseases, type 2 diabetes, and cancer) [6,36,37]. In this context, the EAT-Lancet commission and the Dutch Health Council recommend to consume ≥60% plant-based protein to achieve the future climate goals [[3], [4], [5], [6], [7]]. Therefore, it is important to determine how predominantly plant-based diets can still provide high-quality protein and to identify food groups and combinations that best support amino acid adequacy.

To provide practical insights for dietary transitions, this study assessed the protein content, quality, and sources of predominantly plant-based meals and diets (≥60% plant-based protein) among Dutch adults aged 18 to 79 y, using data from the Dutch National Food Consumption Survey (DNFCS) 2019–2021.

## Methods

### Study design

This observational study used data from the DNFCS 2019–2021, a periodic cross-sectional survey conducted by the National Institute for Public Health and the Environment (RIVM) [38]. Participants were recruited by a market research agency (Kantar) through stratified random sampling based on age, sex, region, urbanization, and educational level, ensuring a representative sample of the Dutch population. Recruitment was conducted in 4-wk cycles. Sampling targets were adjusted across successive waves to preserve population representativeness. A total of 9701 individuals aged 1 to 79 y were invited, of whom 3570 participated. Of those invited, 105 were ineligible, 3540 could not be contacted, 2062 declined participation, and 433 did not complete both 24-h recalls. In addition, two sets of 24-h recalls were deemed unreliable and excluded from analysis. The recruitment goal was to include ∼260 participants in each age (18–50, 51–64, 65–79 y) by sex (males and females) stratum. This target was met for all strata except males aged 51 to 64 y (*n* = 251). Response rates were higher among participants with higher educational attainment (46%) than among those with lower educational attainment (23%). Response was broadly comparable across regions and levels of urbanization. For the present analysis, only data from participants aged 18 to 79 y were used (*n* = 1747), with a total of 9933 main meals. Analyses were conducted without applying survey weights, and results therefore reflect the study sample rather than population-representative estimates. In addition to food intake, self-reported data were collected on sociodemographic characteristics, anthropometrics, and lifestyle factors. The study protocol was reviewed by the Utrecht University Medical Ethical Review Committee, which concluded that it was not subject to the Medical Research Involving Human Subjects Act (WMO) (reference: 19-145/C). We used the STROBE-nut checklist when writing our report [39].

### Dietary intake

#### Study design DNFCS 2019–2021

Food consumption data were collected using two nonconsecutive 24-h dietary recalls per participant, administered within a 2-wk to 6-wk interval, and conducted according to the GloboDiet methodology [40]. Participants aged 18 to 69 y completed both recalls via telephone. For participants aged ≥70 y, the first interview was conducted during a home visit and the second by telephone. During periods with COVID-19 restrictions, both interviews were conducted via telephone. Before each interview, participants aged ≥70 y were asked to complete a structured food diary on two specified days. These diaries were used as memory aids during the 24-h recalls but were not processed as standalone intake data. Dietary recalls were conducted across all seasons and days of the week to ensure representativeness. Participants self-identified their food consumption occasions, which were categorized into three main meals (breakfast, lunch, and dinner) and four in-between moments (before breakfast, morning, afternoon, and evening/night).

#### Dietary intake and amino acid composition

This study calculated the nutritional intake with the Dutch food composition database (NEVO-online 2023) [41]. The data of the DNFCS was based on NEVO-online 2021, whereby codes that were missing were matched to NEVO-online 2023. Amino acid data are not yet available in the NEVO database. Therefore, we extended the NEVO food composition database 2023 with amino acid values for all food products, based on the English (McCance and Widdowson) [42] and Danish (Frida) [43] composition tables. To ensure appropriateness at the product level, NEVO food items were linked as closely as possible to equivalent items in the McCance and Widdowson database. For NEVO food items without a clear equivalent in McCance and Widdowson, we applied a hierarchical approach: *1*) selecting a basic food based on the dominant protein source; *2*) selecting the most comparable food item to obtain an amino acid profile; *3*) consulting the Frida food composition database to retrieve amino acid information; or *4*) constructing a recipe. Furthermore, we added protein digestibility values [44] to obtain a complete NEVO database including amino acid composition and digestibility for all NEVO food products. A targeted literature search identified fecal protein digestibility values for comparable foods, which were used to derive a single digestibility factor for each NEVO food group.

The meal protein quality score (MPQS) [45] was used to assess the protein quality of individual meals in this study. It is a composite meal-level score that integrates total protein, protein digestibility [44], amino acid composition, and amino acid requirements for adults (expressed as milligram essential amino acid per gram protein) [8,13]. The MPQS incorporates individualized essential amino acid requirements per meal to match the body’s metabolic needs. These requirements are derived from a per-meal protein target of 0.3 g/kg and the WHO amino acid reference patterns [8,13]. Specifically, the personalized requirement is the per-meal requirement for each essential amino acid (mg/meal), computed as: 0.3 g protein/kg body weight per meal × WHO reference pattern (mg essential amino acid/g protein), yielding mg essential amino acid per meal for each participant. For protein quantity, we applied a target of 0.3 g/kg body weight per eating occasion, as specified in the original MPQS method [45]. This per-meal target was chosen based on evidence that ∼0.3 g/kg per meal is sufficient to stimulate muscle protein synthesis [21] and, when distributed over three main meals plus snacks, corresponds to an overall intake of ∼1.0 to 1.2 g/kg/d, in line with recommendations for healthy aging [2,[14], [15], [16]]. The MPQS [45] is calculated as:

$$\text{MPQS}={\mathrm{MIN}\left(\%\right)}_{i}\;\left(\frac{\sum_{j}\left[\text{intake}\;\left(\text{mg}\right)\;\text{of}\;{\text{EAA}}_{i}\;\text{from}\;{\text{food}}_{j}\times \text{digestibility}\;\text{factor}\;\text{of}\;{\text{food}}_{j}\right]}{\text{personalised}\;\text{requirement}\;\left(\text{mg}\right)\;\text{of}\;{\text{EAA}}_{i}}\right)$$

EAA, essential amino acid.

As the MPQS is determined by the most limiting essential amino acid according to the WHO reference pattern (i.e., the lowest amino acid score), the scores range from 0, when ≥1 essential amino acid is entirely absent, to 100, where all essential amino acids meet their targets. Values can exceed 100 if every essential amino acid surpasses its requirement.

Total dietary intake was analyzed using a syntax developed by the Amsterdam University of Applied Sciences in R version 4.5.1. (RStudio. Posit Software, 2025). Dietary recall data were analyzed in two ways. First, at the individual level, intake was calculated as the mean of the two recalls per participant. Second, at the meal level, intake was calculated for each reported meal, with meals treated as individual observations. Analyses included total, plant-based, and animal-based protein intake per meal and per day, protein quality (MPQS), and the contribution of various food groups to protein intake. The proportion of meals with suboptimal protein quality (MPQS <100) was examined in relation to both total protein intake and the proportion of plant-based protein. In addition, food group contributions to protein intake per meal were analyzed for meals meeting both protein quantity (≥20 g) and quality (MPQS ≥100) thresholds, comparing those with ≥60% compared with <60% plant-based protein intake following the recommendations of policy makers and health councils [4,5,17].

### Statistical analysis

All analyses were performed using R version 4.5.1. Descriptive statistics were used to summarize the characteristics of participants included in the DNFCS. Continuous variables were presented as mean ± SD or median (IQR), depending on the distribution of the data. Categorical variables were summarized as frequencies and percentages.

Normality of continuous variables was assessed using histograms and the Shapiro–Wilk test. For comparisons between two groups, normally distributed continuous variables were analyzed using independent *t*-tests, whereas non-normally distributed variables were analyzed using the Wilcoxon rank-sum test. Categorical variables were compared using chi-squared tests (expected cell count ≥5) or Fisher’s exact test (expected cell count <5).

For comparisons across four protein intake groups (<0.66, 0.66–0.82, 0.83–1.19, ≥1.2 g/kg/d), one-way analysis of variance (ANOVA) (normally distributed data) or Kruskal–Wallis tests (non-normally distributed data) were used for continuous variables. When significant, post hoc pairwise comparisons were performed using Tukey’s test (for ANOVA) or Dunn’s test with Bonferroni correction (*P* = 0.05/number of comparisons) (for Kruskal–Wallis). For significant categorical variables, post hoc Bonferroni-corrected pairwise proportion test (expected cell count ≥5) or Bonferroni-corrected pairwise Fisher’s exact test (expected cell count <5) were conducted.

Pearson’s correlation coefficient was used to assess the associations between total protein intake and protein quality, as well as between the proportion of plant-based protein and protein quality. For all analyses, statistical significance was defined as *P* < 0.05.

## Results

A total of 1747 adults were included in our analyses. The participants had a median (IQR) age of 57 (44–68) y and 50% were male. The median BMI was 25.7 (23.3–29.3) kg/m^2^. Most of the participants were White (93%), and 25% were low educated (Table 1).

**TABLE 1:** Population characteristics

	Total	Plant-based protein		*P* value^1^
		<60%	≥60%	
*n*	1747	1600	147	—
Females [*n* (%)]	867 (50)	782 (49)	85 (58)	0.047
White [*n* (%)]	1623 (93)	1498 (94)	125 (85)	<0.001
Initial education^2^ [*n* (%)]	445 (25)	429 (27)	16 (11)	<0.001
Age (y)	57.0 (44.0–68.0)	58.0 (45.0–68.0)	48.0 (35.5–61.0)	<0.001
Weight (kg)	80.0 (70.0–90.0)	80.0 (70.0–91.0)	73.0 (63.5–83.5)	<0.001
Height (cm)	175 (168–182)	175 (168–182)	174 (166–181)	0.438
BMI (kg/m^2^)	25.7 (23.3–29.3)	26.0 (23.6–29.4)	23.3 (21.6–25.8)	<0.001
Protein (g/d)	73.4 (60.5–88.9)	74.8 (61.8–90.2)	61.3 (46.9–74.7)	<0.001
Protein (g/kg/d)	0.93 (0.75–1.13)	0.94 (0.75–1.14)	0.83 (0.63–1.05)	<0.001
Energy intake (kcal/d)	1949 (1619–2331)	1954 (1627–2333)	1847 (1572–2327)	0.286

Groups are based on the proportion of plant-based protein.

Data are presented as median (IQR) or *n* (%).

1 Differences between two groups were tested using a Wilcoxon rank-sum test (continuous variables) or chi-squared test (categorical variables).

2 Initial education is defined as community college or less educated (primary and secondary education).

Participants with ≥60% plant-based protein intake had a lower body weight and BMI (both *P* < 0.001). They were also younger (*P* < 0.001), more often female (*P* = 0.047), more often non-Dutch (*P* < 0.001), and more often having completed higher education (*P* < 0.001).

As protein intake increased across the four intake categories (<0.66, 0.66–0.82, 0.83–1.19, and ≥1.2 g/kg/d), body weight and BMI significantly decreased, whereas energy intake significantly increased, both in the total population and in the ≥60% plant-based protein group (Supplementary Tables 1–3). Median daily energy intake in the ≥60% plant-based protein group was 1523 (1255–1720) kcal in the <0.66 g/kg/d group, 1694 (1497–2132) kcal in the 0.66–0.82 g/kg/d group, 2113 (1774–2418) kcal in the 0.83 to 1.19 g/kg/d group, and 2663 (2271–2992) kcal ≥1.2 g/kg/d, with similar intakes observed in the total population.

### Protein intake

The median protein intake of the total population was 73.4 (60.5–88.9) g/d or 0.93 (0.75–1.13) g/kg/d. In this population, 8% (*n* = 147) had a diet comprising ≥60% plant-based protein, with a significantly lower median protein intake of 61.3 (46.9–74.7) g/d or 0.83 (0.63–1.05) g/kg/d compared with individuals consuming <60% plant-based protein [74.8 (61.8–90.2) g/d or 0.94 (0.75–1.14) g/kg/d; *P* <0.001]. Among all participants, 16% (*n* = 278) had a protein intake <0.66 g/kg/d, 19% (*n* = 334) between 0.66 and 0.82 g/kg/d, 46% (*n* = 803) between 0.83–1.19 g/kg/d, whereas 19% (*n* = 332) met or exceeded the intake of ≥1.2 g/kg/d. Within the subgroup consuming ≥60% plant-based protein, 29% (*n* = 43) had a protein intake <0.66 g/kg/d, 18% (*n* = 27) between 0.66 and 0.82 g/kg/d, 41% (*n* = 60) between 0.83 and 1.19 g/kg/d, and 12% (*n* = 17) reached an intake of ≥1.2 g/kg/d. The proportion of adults with a protein intake below the EAR (<0.66 g/kg/d) was significantly higher in those consuming ≥60% plant-based protein (29%) compared with those consuming <60% plant-based protein (15%; *P* < 0.001).

**FIGURE 1:**
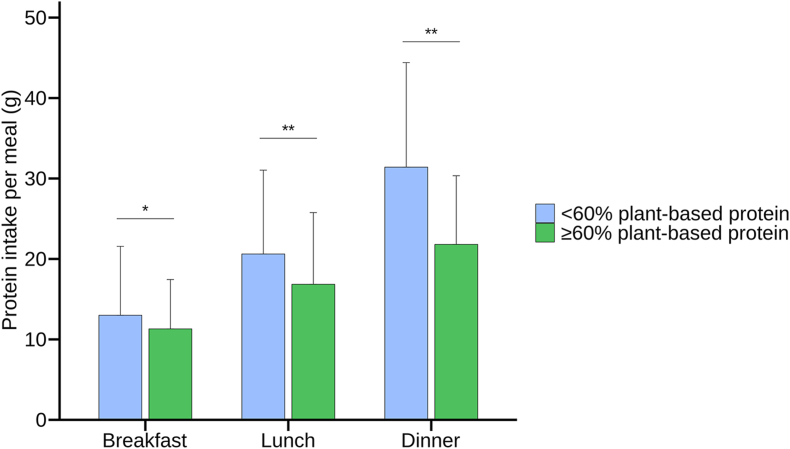
Protein intake (g ± SD) per meal. Differences between two groups were tested using an independent *t-*test. ∗*P* value <0.01, ∗∗*P* value <0.0001.

Protein intake was significantly lower across all meals in individuals consuming ≥60% plant-based protein compared with those consuming <60% plant-based protein (Figure 1). Regardless of plant-based protein intake, dinner contributed most of the protein, followed by lunch and breakfast. This pattern was also observed across the four protein intake subcategories of <0.66, 0.66 to 0.82, 0.83 to 1.19, and ≥1.2 g/kg/d (Supplementary Figure 1–3).

Among participants with ≥60% plant-based protein intake, 72.2% had ≥1 meal and 22.9% had ≥2 meals providing ≥20 g of protein, which was considerably lower than the 91.4% and 49.0%, respectively, among those with <60% plant-based protein. The percentage of participants meeting ≥20 g protein across all three meals was low in both groups: 2.1% for the ≥60% plant-based protein group compared with 6.7% for the <60% plant-based protein group. Dinner most often contained ≥20 g protein in both individuals consuming ≥60% plant-based protein (61.9%) and in the total population (83.2%) compared with breakfast and lunch. The median protein intake of dinner was 21.9 (16.1–27.4) g in the ≥60% plant-based protein group, and 29.8 (22.5–38.4) g in the <60% plant-based protein group.

### Protein quality

**FIGURE 2:**
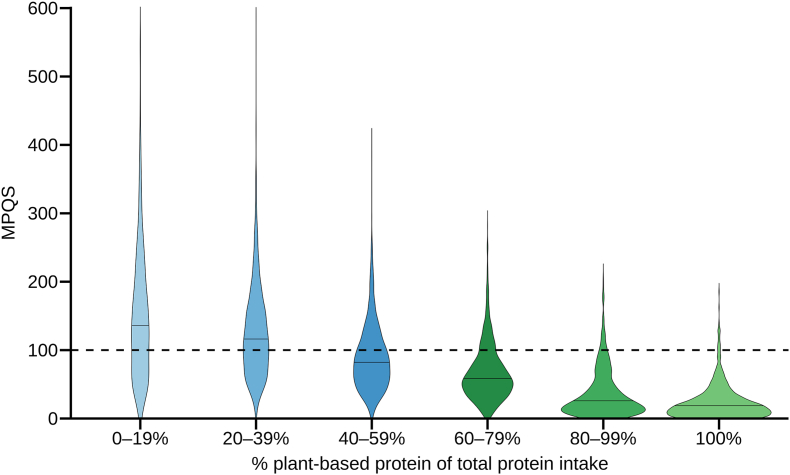
Meal Protein Quality Score (MPQS) distributions by plant protein proportion of total protein for breakfast, lunch, and dinner. Lines in the plots indicate the median, the dashed line indicates an MPQS of 100. Sample sizes (meals) per category: 0%–19% (*n* = 1626); 20%–39% (*n* = 3090); 40%–59% (*n* = 2485); 60%–79% (*n* = 1167); 80%–99% (*n* = 664); and 100% (*n* = 843).

Figure 2 illustrates the distribution of MPQS, a measure of protein quality per meal, across increasing proportions of plant-based protein intake. Consuming ≥60% plant-based protein resulted in a significant lower median MPQS [34.3 (15.4–64.4)] compared with consuming <60% plant-based protein [105.0 (66.5–155.2); *P* < 0.001]. Ten percent (269) of meals with ≥60% plant-based protein achieved an adequate MPQS (≥100).

Across all meals regardless of plant protein intake with suboptimal protein quality (MPQS <100), the most frequently limiting essential amino acid was lysine (62%), followed by methionine (17%), leucine (13%), valine (3%), isoleucine (3%), and cysteine (2%). Among meals containing ≥60% plant-based protein and an MPQS <100, lysine was the limiting amino acid in 78% of cases, followed by methionine (13%), leucine (5%), isoleucine (3%), valine (0.5%), and cysteine (0.5%). In 100% plant-based (vegan) meals with MPQS <100, lysine remained most frequently limiting (59%), followed by methionine (21%), isoleucine (11%), and leucine (9%).

### Protein quantity and quality

**FIGURE 3:**
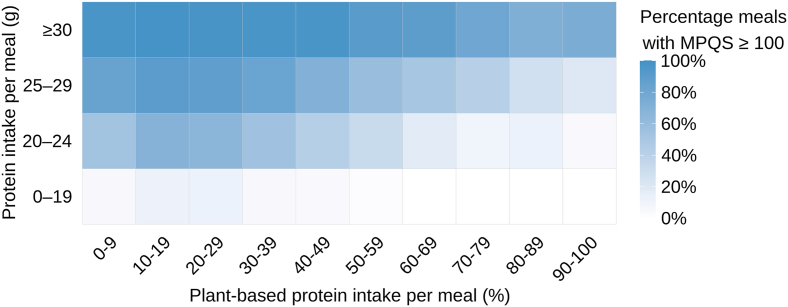
Heatmap with the proportion of meals that have an adequate protein quality (MPQS ≥100). MPQS, Meal Protein Quality Score.

The heatmap (Figure 3) presents the proportion of meals achieving an adequate protein quality (MPQS ≥100), stratified by protein intake and the proportion of plant-based protein per meal. As expected, MPQS was lower in meals providing <20 g of protein, reflecting the impact of protein quantity on protein quality per meal. Meals with higher protein content generally achieved a higher MPQS (*r* = 0.90, *P* <0.001). Meanwhile, meals with higher proportions of plant-based protein result in lower protein quality (*r* = −0.53, *P* < 0.001).

### Food groups

**FIGURE 4:**
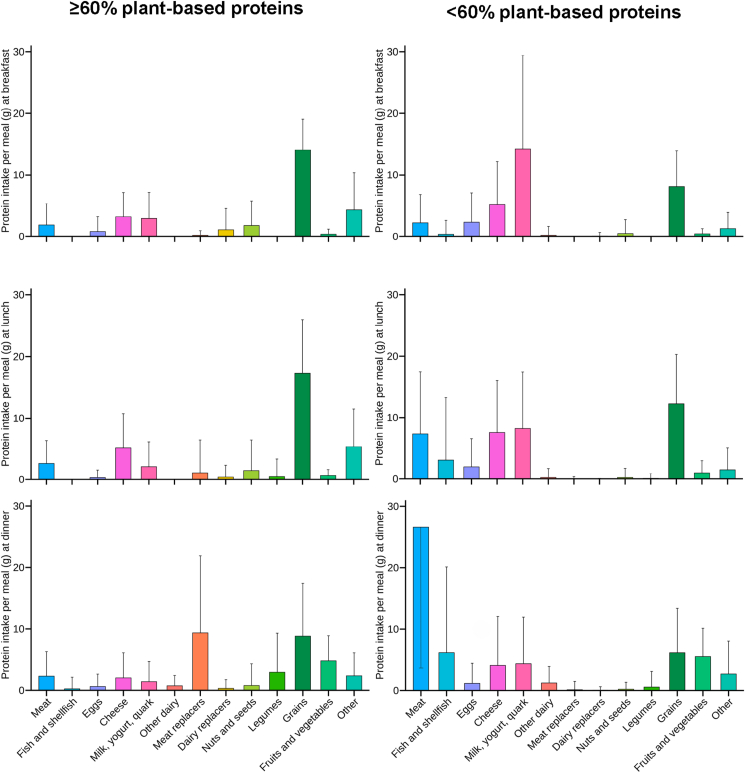
Protein intake (g ± SD) from food groups in individuals consuming breakfast, lunch, and dinner meals with ≥20 g total protein, MPQS ≥100, and ≥60% plant-based protein (left bar charts) or <60% plant-based protein (right bar charts). Meat and dairy alternatives include processed plant-based products replacing meat or dairy (e.g., plant-based burgers, milk, yogurt); legumes and nuts were not classified as meat alternatives. MPQS, Meal Protein Quality Score.

Protein intake from food groups consumed by individuals whose meals provided ≥20 g protein, ≥60% plant-based protein, and optimal protein quality (MPQS ≥100) were assessed (Figure 4). These criteria were achieved in 3% (265) meals: 1% (32) breakfast, 3% (107) lunch, and 4% (126) dinner meals. Those who met these criteria primarily consumed protein from grains (46%, 14 g), cheese (11%, 3 g), and milk, yogurt, and quark (10%, 3 g) at breakfast. At lunch, the main protein sources were grains (47%, 17 g), cheese (14%, 5 g), and meat (7%, 3 g). Protein intake at dinner was primarily derived from meat alternatives (26%, 9 g), grains (24%, 9 g), and fruits and vegetables (13%, 5 g).

Protein intake from food groups consumed by individuals whose meals provided ≥20 g protein, <60% plant-based protein, and optimal protein quality (MPQS ≥100) were also assessed. These criteria were achieved in 36% (3582) meals: 10% (337) breakfast, 36% (1130) lunch, and 62% (2115) dinner meals. Those who met these criteria primarily consumed protein from milk, yogurt, and quark (41%, 14 g), grains (23%, 8 g), and cheese (15%, 5 g) at breakfast. Protein intake at lunch was primarily derived from grains (28%, 12 g), milk, yogurt, and quark (19%, 8 g), cheese (18%, 7 g), and meat (17%, 7 g). At dinner, the main protein sources were meat (45%, 27 g), (shell)fish (11%, 6 g), grains (10%, 6 g), and fruits and vegetables (9%, 6 g).

## Discussion

This study examined protein intake, quality, and sources in predominantly plant-based meals and diets (≥60% plant-based protein) among Dutch adults aged 18 to 79 y. Only 8% of the population consumed ≥60% plant-based protein, with a significantly lower protein intake and protein quality compared with those consuming <60% plant-based protein. In meals with ≥60% plant-based protein and suboptimal protein quality (MPQS <100), lysine was the most frequently limiting amino acid. We found that as the proportion of plant-based protein increased, both total protein quantity and quality decreased. Only 3% of all main meals provided ≥20 g of protein, consisted of ≥60% plant-based protein, and achieved optimal protein quality (MPQS ≥100). These meals typically derive their protein from grains and dairy at breakfast and lunch, and from grains and meat alternatives at dinner.

### Protein intake

Our findings indicate that protein intake in the Dutch adult population does not fully align with current dietary recommendations. A subset of the study population consumed protein below the EAR (0.66 g/kg/d), suggesting a potential risk of inadequacy. This risk was more prevalent among individuals whose diets consisted of ≥60% plant-based protein compared with those with <60% plant-based protein. Furthermore, a substantial proportion did not reach the RDA (0.83 g/kg/d), particularly among those consuming ≥60% plant-based protein. Notably, only 19% of the total population and even fewer individuals consuming ≥60% plant-based protein achieved an intake of ≥1.2 g/kg/d, a level often recommended for older adults, individuals with high levels of physical activity, and those with acute or chronic disease [2,14]. Similarly, a recent study among hospitalized patients, whose diets consisted of 31% plant-based protein, reported that 80% did not reach the recommended protein intake of ≥1.2 g/kg/d [46]. However, our sample also included healthy younger adults, and we did not specifically assess athletes or clinical populations. Thus, ≥1.2 g/kg/d should be viewed as a reference point, most relevant for older adults and others with elevated protein needs. These findings highlight not only the prevalence of suboptimal intake relative to current reference values but also the limited proportion of individuals achieving protein levels considered beneficial for specific populations with higher requirements, which is especially challenging when consuming more plant-based protein.

Lower protein intake in more plant-based diets aligns with previous findings [28,29] and can be attributed to the generally lower protein density of plant foods per portion [47]. Within the total population, and especially among individuals consuming predominantly plant-based protein, protein intake was lowest at breakfast and progressively higher at lunch and dinner, mirroring patterns observed in earlier studies [22,23,48]. In meals with ≥60% plant-based protein, average protein intake at both breakfast and lunch remained <20 g, and only 2.1% of participants consumed ≥20 g of protein at all three meals. Only dinner in the <60% plant-based group reached on average the ≥25 to 30 g protein threshold, a level associated with optimal stimulation of muscle protein synthesis in older adults [[19], [20], [21]]. These findings highlight a potential risk of insufficient protein intake per day and per meal, particularly in those with elevated needs.

### Protein quality

In addition to low protein intake, diets with a high proportion of plant-based protein often showed reduced protein quality, consistent with previous research [[45], [46], [47]]. Among meals with ≥60% plant-based protein, only 10% achieved optimal protein quality (MPQS ≥100), suggesting that amino acid availability and protein utilization may be compromised. Within meals with ≥60% plant-based protein in our study, lysine was the most frequently limiting essential amino acid, followed by methionine, leucine, and isoleucine. A similar pattern was observed in two recent studies, which identified particularly lysine as the most limiting amino acid in meals containing 65% to 99% plant-based protein [45] and meals containing ≥50% plant-based protein [46]. In our study population, the low lysine intake can be attributed to the inherently limited lysine content of cereal grains [9], which constituted a major source of protein in diets containing ≥60% plant-based protein.

Our findings emphasize that achieving adequate protein quality requires a minimum protein intake of ∼0.3 g/kg per meal, consistent with the assumption underlying the MPQS [45]. The MPQS would, by definition, change if a different per-meal protein target were applied. Evidence suggests that the per-meal protein intake required to maximize muscle protein synthesis varies by age and physiological state (e.g., ∼0.24 g/kg in younger adults, ∼0.31 g/kg during recovery from resistance exercise, and ∼0.40 g/kg in older adults) [15,18]. Given that our study population ranged from 18 to 79 y, we used 0.3 g/kg per meal as a reasonable intermediate value and to remain consistent with the original MPQS approach [45]. In addition to protein quantity per meal, the composition of protein sources also plays a crucial role in determining overall quality. Meeting this per-meal protein threshold appears more challenging with increasing proportions of plant-based protein than with animal-based protein, as lower quality protein may further reduce essential amino acid availability [29,45]. The findings highlight the importance of prioritizing adequate protein intake when following more plant-based diets. At lower protein intakes, combining complementary protein sources is essential to ensure sufficient amino acid availability.

### Food groups

Combining all protein-related and environmentally sustainable dietary recommendations [[3], [4], [5], [6]], including consuming a sufficient protein quantity (≥20 g), high protein quality (MPQS ≥ 100), and ensuring that ≤60% of the protein is plant-based, appears to be particularly challenging in practice. In the study population, only 3% of all meals met all three criteria. Although these represent a relatively small proportion of total meals, the findings demonstrate that it is feasible to prepare largely plant-based meals that provide adequate protein quantity and quality. In these meals in the present study, grains and dairy were the main sources of protein at breakfast and lunch, whereas grains and meat alternatives predominated at dinner. Because many plant-based foods have suboptimal protein quality, combining complementary plant protein sources can improve overall protein adequacy and ensure a more balanced amino acid profile [10,47]. According to these two studies, blending protein with low lysine and high methionine content (such as corn, hemp, or brown rice) with those rich in lysine but lower in methionine (such as soy or pea) yields a more complete amino acid composition. Such combinations require ∼1.1 to 1.9 times more total protein to achieve amino acid adequacy than high-quality protein, compared with 2 to 4 times when relying on a single plant protein source [47]. However, achieving these complementary combinations in everyday diets may be challenging, particularly at breakfast and lunch. This likely reflects Western eating patterns, where meals often rely on a limited number of staple foods and may not consistently provide optimal amino acid balance. In our data, plant protein complementarity at breakfast was virtually absent because legumes and nuts and seeds contributed only a small share of protein intake, limiting opportunities for within-meal complementarity, and this pattern may also apply to other Western dietary settings. In theory, very large portions (3–8 times) of grains such as wheat or oats would be needed to compensate for their low lysine and methionine content [10,47]. Beyond being unrealistic, such a strategy is also undesirable because the chronic disease benefits associated with higher plant protein intake appear to depend on overall diet quality and the types of plant protein sources consumed [49]. In our population, plant protein was largely derived from cereal-based staples, whereas legumes, nuts and seeds contributed relatively little, which may limit both opportunities for amino acid complementarity and broader nutritional benefits. By contrast, adding small amounts of animal-based foods can markedly improve amino acid adequacy and reduce the total protein required, ∼1.05 to 1.4 times the amount of high-quality protein blends would suffice. Consistent with our data, practical examples include combining grains with dairy at breakfast or lunch (e.g., bread with cheese and milk or yogurt with cereals) and combining grains with plant-based meat alternatives at dinner (e.g., pasta or potatoes with soy-based meat alternatives).

### Strengths and limitations

Strengths of our study include the use of a nationally representative and relatively large dataset, a focus on protein quality in addition to quantity, and the detailed examination of meals with >60% plant-based protein. However, a limitation is that only a small proportion of the population consumed diets with >60% plant-based protein, resulting in a limited sample size for this group. Moreover, although higher protein requirements are particularly relevant for individuals with increased needs, such as older adults, this study included a broader population of adults aged 18 to 79 y and is therefore not specifically representative of older adults alone. Furthermore, as analyses were unweighted, the findings may not fully represent the Dutch adult population, particularly with respect to sociodemographic characteristics. In addition, our analysis focused solely on main meals, whereby protein intake from between-meal occasions was not included in the assessment of protein quality. Although it is generally accepted that protein should be consumed within a specific timeframe to effectively stimulate muscle protein synthesis [50,51], the exact duration of this anabolic window remains uncertain. As such, the timeframe in this analysis may have been too narrow. This may have led to an underestimation of the protein quality, particularly in more plant-based meals. In addition, 24-h recalls may misestimate individuals’ habitual protein intake, although they are generally considered appropriate for estimating intake at the group level [52]. Another limitation is that digestibility varies across foods and processing methods. Applying a single digestibility factor per NEVO food group may therefore misclassify digestibility for specific items and could lead to underestimation or overestimation of digestible protein and amino acid intake for some foods. In addition, digestibility data for dairy and meat replacers are limited, which adds uncertainty to estimates for these product categories.

In conclusion, this study demonstrates that only a small proportion of Dutch adults currently meet both protein-related recommendations and sustainability goals. Diets and meals with a higher proportion of plant-based protein had lower protein quantity and quality, highlighting a potential nutritional challenge in the shift toward more sustainable eating patterns. In particular, ensuring adequate protein quality is crucial when total protein intake is lower and in predominantly plant-based diets, as plant-based protein sources generally have lower digestibility and an incomplete amino acid profile. These findings underscore the importance of nutritional guidance, especially for individuals with higher protein needs, such as older adults and (hospitalized) patients, to support a successful and nutritionally adequate transition toward more plant-based dietary patterns.

## Author contributions

The authors’ responsibilities were as follows – SLVO: conducted the data analysis; and all authors: designed the research, wrote the manuscript, and read and approved the final manuscript.

## Data availability

The data described in this manuscript are publicly available from the Dutch Health Council (RIVM) and cannot be redistributed by the authors. The dataset can be requested via the RIVM website: https://www.wateetnederland.nl/aanvraagfomulier-gebruik-dataset-vcp. The codebook and analytic code are publicly available without restriction at https://doi.org/10.21943/auas.30731549.

## Declaration of generative AI in scientific writing

During the preparation of this work, the authors used ChatGPT (AI language model) to help with grammar, spelling, and sentence structure. No analytical or interpretative content was produced by the model. After using this model, the authors reviewed and edited the content as needed and take full responsibility for the content of the publication.

## Funding

This project was supported by Top Consortia for Knowledge and Innovation (TKI) (LWV21.57), which is a public-private partnership. The researchers had full responsibility for study design, data collection, analysis, interpretation, writing of the manuscript, and the decision to submit for publication.

## Conflict of interest

The authors declare no competing interests.

## Figures and Tables

**Figure:**
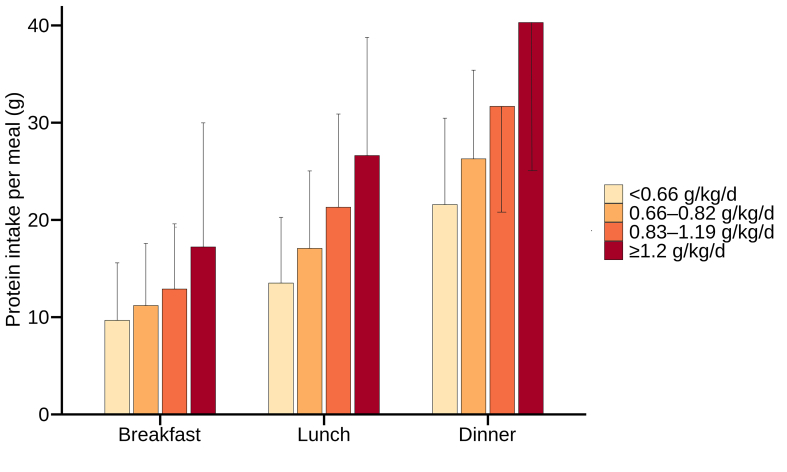


**Figure:**
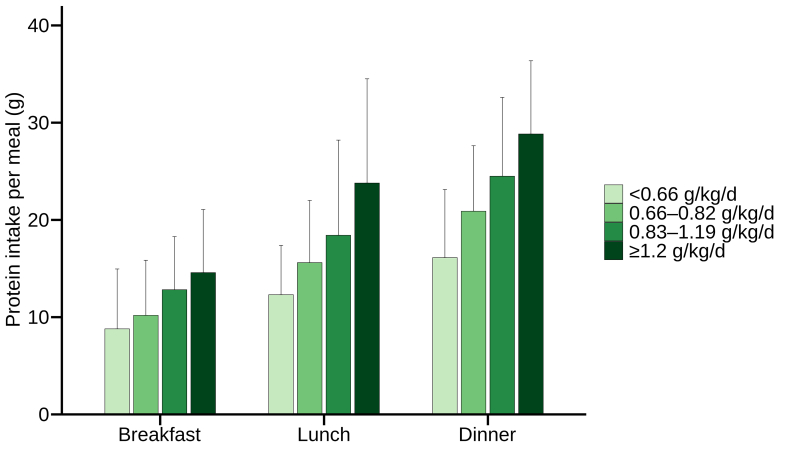


**Figure:**