# Supplementary material for: Differences in Protein Quantity and Quality Across a Spectrum of Plant-Based Meals: Analysis of a Large National Dietary Survey
Source: Curr Dev Nutr. 2026 Jan 22;10(2):107641. doi: 10.1016/j.cdnut.2026.107641 (PMC12925101; doi:10.1016/j.cdnut.2026.107641)
Supplement: Multimedia component 1 [file mmc1.docx]

## Supplements

**Supplementary table 1** | Population characteristics total population (n=1747)

|  | **<0.66** | **0.66-0.82** | **0.83-1.19** | **≥1.2** | ***p*^1^** |
| --- | --- | --- | --- | --- | --- |
| n | 278 | 334 | 787 | 348 |  |
| Women, *n* (%) | 160 (58)^a^ | 181 (54) | 373 (47) | 153 (44)^b^ | 0.001 |
| White, *n* (%) | 246 (88)^a^ | 306 (92) | 747 (95)^b^ | 324 (93) | 0.003 |
| Initial education^2^, *n* (%) | 76 (27) | 100 (30)^a^ | 203 (26) | 66 (19)^b^ | 0.002 |
| Age, years | 56.0 [45.0-66.0] | 57.0 [45.0-68.0] | 59.0 [45.0-68.0] | 56.0 [41.8-67.0] | 0.162 |
| Weight, kg | 92.0 [80.0-105.0]^a^ | 86.0 [75.0-95.0]^b^ | 78.0 [69.0-88.0]^c^ | 72.0 [62.8-80.0]^d^ | <0.001 |
| Height, cm | 175 [167-180] | 173 [168-182] | 175 [168-182] | 174 [168-182] | 0.515 |
| BMI, kg/m^2^ | 30.1 [26.3-35.1]^a^ | 28.0 [24.8-31.1]^b^ | 25.3 [23.2-27.8]^c^ | 23.4 [21.3-25.2]^d^ | <0.001 |
| Protein, g/d | 51.7 [43.4-59.4]^a^ | 64.1 [56.4-71.1]^b^ | 77.6 [68.0-87.2]^c^ | 99.2 [86.6-115.0]^d^ | <0.001 |
| Protein, g/kg/d | 0.57 [0.50-0.62]^a^ | 0.75 [0.71-0.78]^b^ | 0.99 [0.90-1.07]^c^ | 1.37 [1.27-1.52]^d^ | <0.001 |
| Energy intake, kcal/d | 1514 [1243.5-1829]^a^ | 1729 [1487-2031]^b^ | 2035 [1756-2319]^c^ | 2440 [2048-2911]^d^ | <0.001 |

Groups are based on protein intake (g/kg/d). Data is presented as median [IQR] or *n* (%). BMI = body mass index. ^1^Differences between the four groups were tested using the Kruskal-Wallis test (continuous variables) or chi-squared test (categorical variables). Different superscript letters indicate significant group differences (*p* < 0.05; Bonferroni-corrected Dunn test for continuous variables or Bonferroni-corrected pairwise test for categorical variables). ^2^Initial education is defined as community college or less educated (primary and secondary education).

**Supplementary table 2** | Population characteristics in ≥60% plant-based protein (n=147)

|  | **<0.66** | **0.66-0.82** | **0.83-1.19** | **≥1.2** | ***p*^1^** |
| --- | --- | --- | --- | --- | --- |
| n | 43 | 27 | 57 | 20 |  |
| Women, *n* (%) | 31 (72) | 16 (59) | 30 (53) | 8 (40) | 0.077 |
| White, *n* (%) | 30 (70)^a^ | 26 (96)^b^ | 53 (93) | 16 (80) | 0.003 |
| Initial education^2^, *n* (%) | 6 (14) | 3 (11) | 5 (9) | 2 (10) | 0.578 |
| Age, years | 49.0 [35.5-61.5] | 47.0 [37.0-59.0] | 53.0 [41.0-61.0] | 37.0 [30.0-55.8] | 0.644 |
| Weight, kg | 80.0 [73.0-90.0]^a^ | 79.0 [61.0-88.0] | 70.0 [62.0-78.0]^b^ | 65.0 [64.0-73.0]^b^ | <0.001 |
| Height, cm | 173 [165-178] | 174 [166-180] | 175 [167-185] | 173 [167-183] | 0.588 |
| BMI, kg/m^2^ | 27.5 [23.2-30.1]^a^ | 24.7 [21.8-26.5] | 22.8 [20.9-24.0]^b^ | 22.6 [21.0-23.1]^b^ | <0.001 |
| Protein, g/d | 45.0 [35.0-53.1]^a^ | 56.6 [44.8-69.6]^b^ | 67.8 [58.6-75.6]^c^ | 89.9 [81.4-98.8]^d^ | <0.001 |
| Protein, g/kg/d | 0.57 [0.47-0.62]^a^ | 0.75 [0.70-0.79]^b^ | 0.96 [0.89-1.06]^c^ | 1.37 [1.25-1.51]^d^ | <0.001 |
| Energy intake, kcal/d | 1523 [1255-1720]^a^ | 1694 [1497-2132]^ab^ | 2113 [1774-2418]^b^ | 2663 [2271-2992]^c^ | <0.001 |

Groups are based on protein intake (g/kg/d). Data is presented as median [IQR] or n (%). BMI = body mass index. ^1^Differences between the four groups were tested using the Kruskal-Wallis test (continuous variables) or chi-squared test (expected cell count ≥5) or Fisher’s exact test (expected cell count <5) (categorical variables). Different superscript letters indicate significant group differences (*p* < 0.05; Bonferroni-corrected Dunn test for continuous variables or Bonferroni-corrected Fisher’s exact test (expected cell count <5) for categorical variables). ^2^Initial education is defined as community college or less educated (primary and secondary education).

**Supplementary table 3** | Population characteristics in <60% plant-based protein (n=1600)

|  | **<0.66** | **0.66-0.82** | **0.83-1.19** | **≥1.2** | ***p*^1^** |
| --- | --- | --- | --- | --- | --- |
| n | 235 | 307 | 730 | 328 |  |
| Women, *n* (%) | 129 (55)^a^ | 165 (54) | 343 (47) | 145 (44)^b^ | 0.017 |
| White, *n* (%) | 216 (92) | 280 (91) | 694 (95) | 308 (94) | 0.080 |
| Initial education^2^, *n* (%) | 70 (30)^a^ | 97 (32)^a^ | 198 (27) | 64 (20)^b^ | <0.001 |
| Age, years | 57.0 [48.0-66.0] | 58.0 [46.0-68.0] | 60.0 [45.0-68.0] | 57.0 [43.8-67.0] | 0.321 |
| Weight, kg | 95.0 [83.5-105.5]^a^ | 86.0 [76.0-95.0]^b^ | 79.0 [70.0-88.0]^c^ | 72.0 [62.0-80.2]^d^ | <0.001 |
| Height, cm | 175 [167-181] | 173 [168-182] | 175 [168-182] | 175 [168-182] | 0.712 |
| BMI, kg/m^2^ | 30.5 [27.1-35.5]^a^ | 28.4 [25.1-31.4]^b^ | 25.5 [23.5-28.1]^c^ | 23.5 [21.4-25.3]^d^ | <0.001 |
| Protein, g/d | 53.1 [44.7-60.1]^a^ | 64.3 [56.8-71.2]^b^ | 78.2 [69.1-88.1]^c^ | 99.8 [87.2-115.3]^d^ | <0.001 |
| Protein, g/kg/d | 0.57 [0.51-0.62]^a^ | 0.75 [0.71-0.78]^b^ | 0.99 [0.90-1.07]^c^ | 1.37 [1.27-1.52]^d^ | <0.001 |
| Energy intake, kcal/d | 1504 [1241-1847]^a^ | 1730 [1488-2023]^b^ | 2028 [1756-2314]^c^ | 2434 [2029-2885]^d^ | <0.001 |

Groups are based on protein intake (g/kg/d). Data is presented as median [IQR] or n (%). BMI = body mass index. ^1^Differences between the four groups were tested using the Kruskal-Wallis test (continuous variables) or chi-squared test (categorical variables). Different superscript letters indicate significant group differences (*p* < 0.05; Bonferroni-corrected Dunn test for continuous variables or Bonferroni-corrected pairwise test for categorical variables). ^2^Initial education is defined as community college or less educated (primary and secondary education).

**
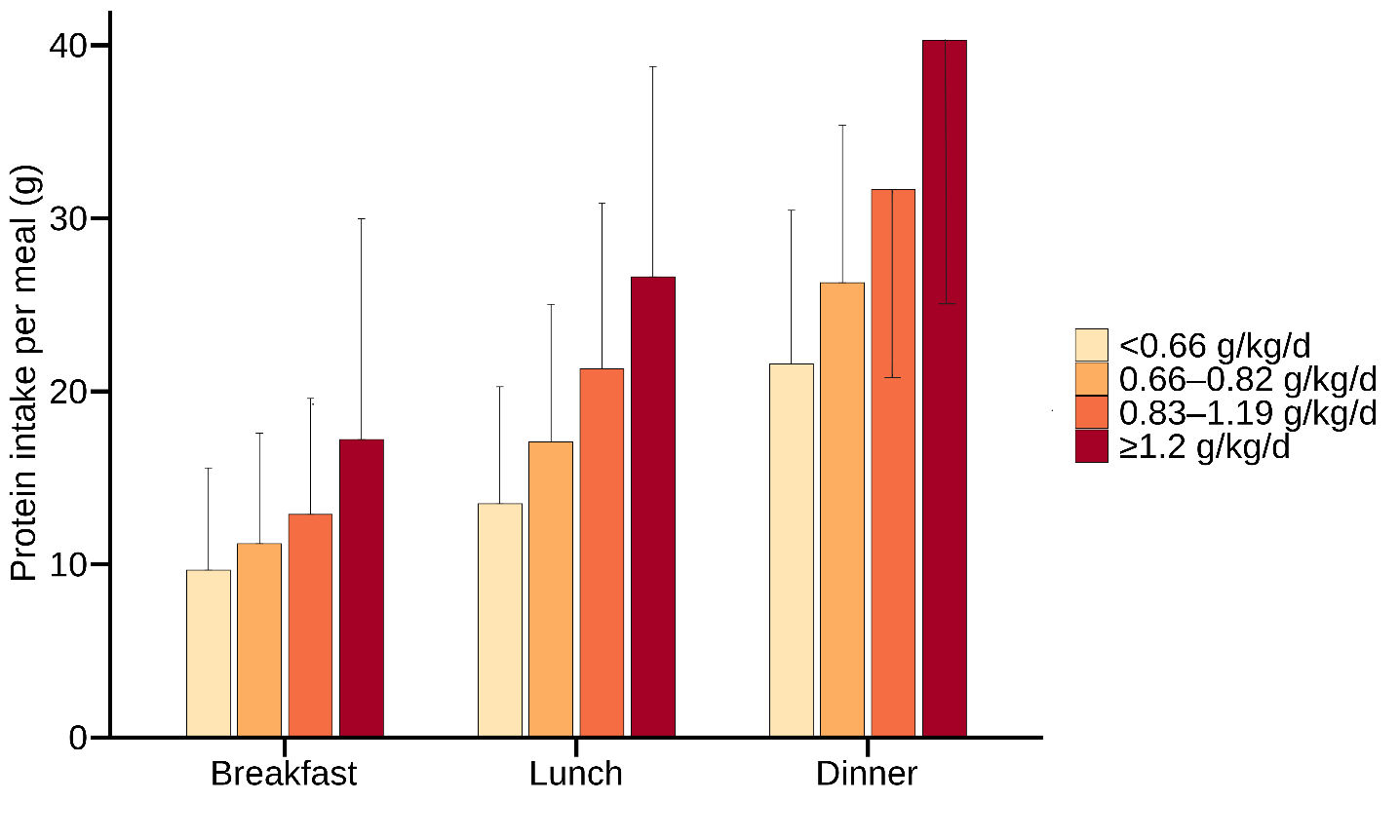
**

**Supplementary figure 1** | Protein intake (g ± SD) per meal across different protein intake groups

**
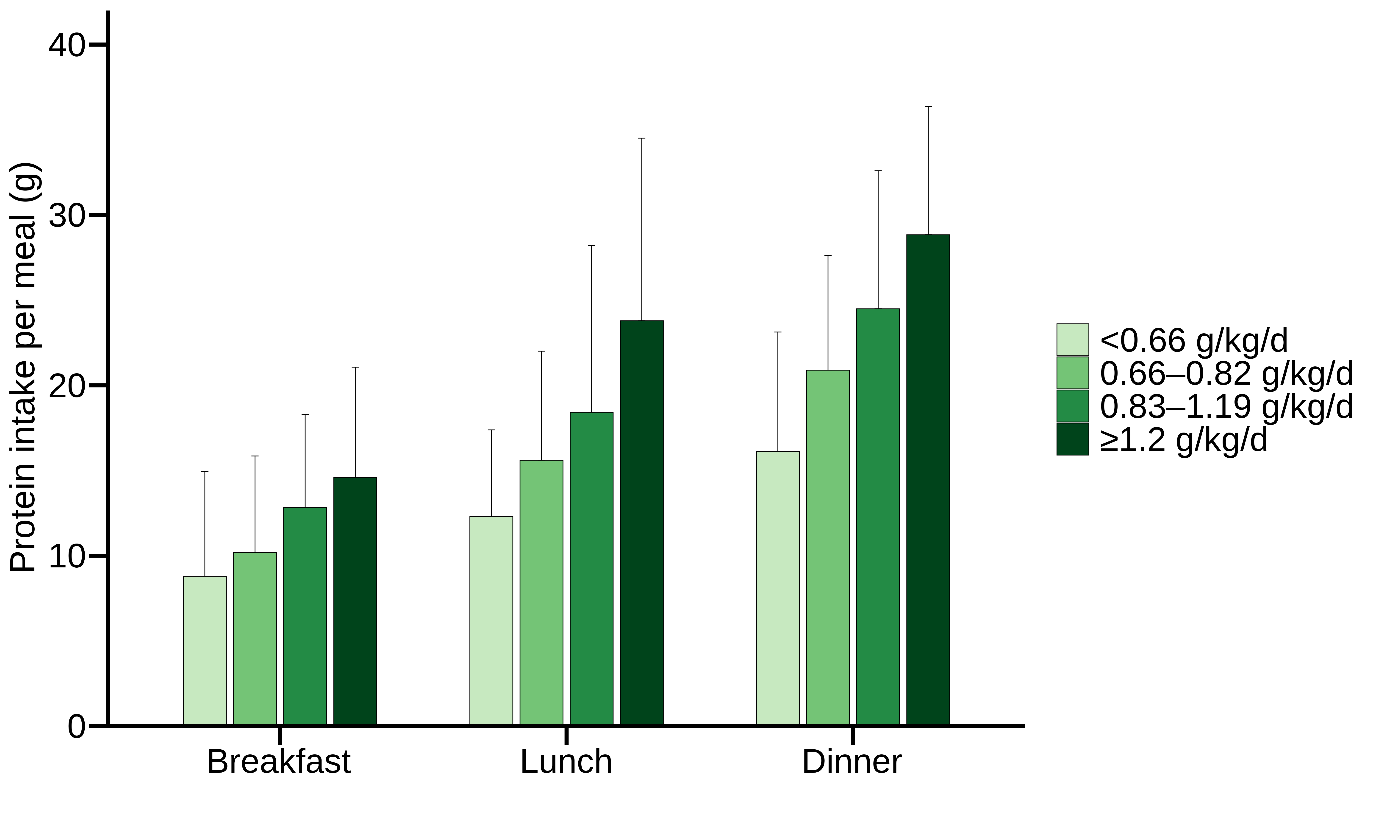
Supplementary figure 2** | Protein intake (g ± SD) per meal across different protein intake groups in participants consuming ≥60% plant-based protein

**
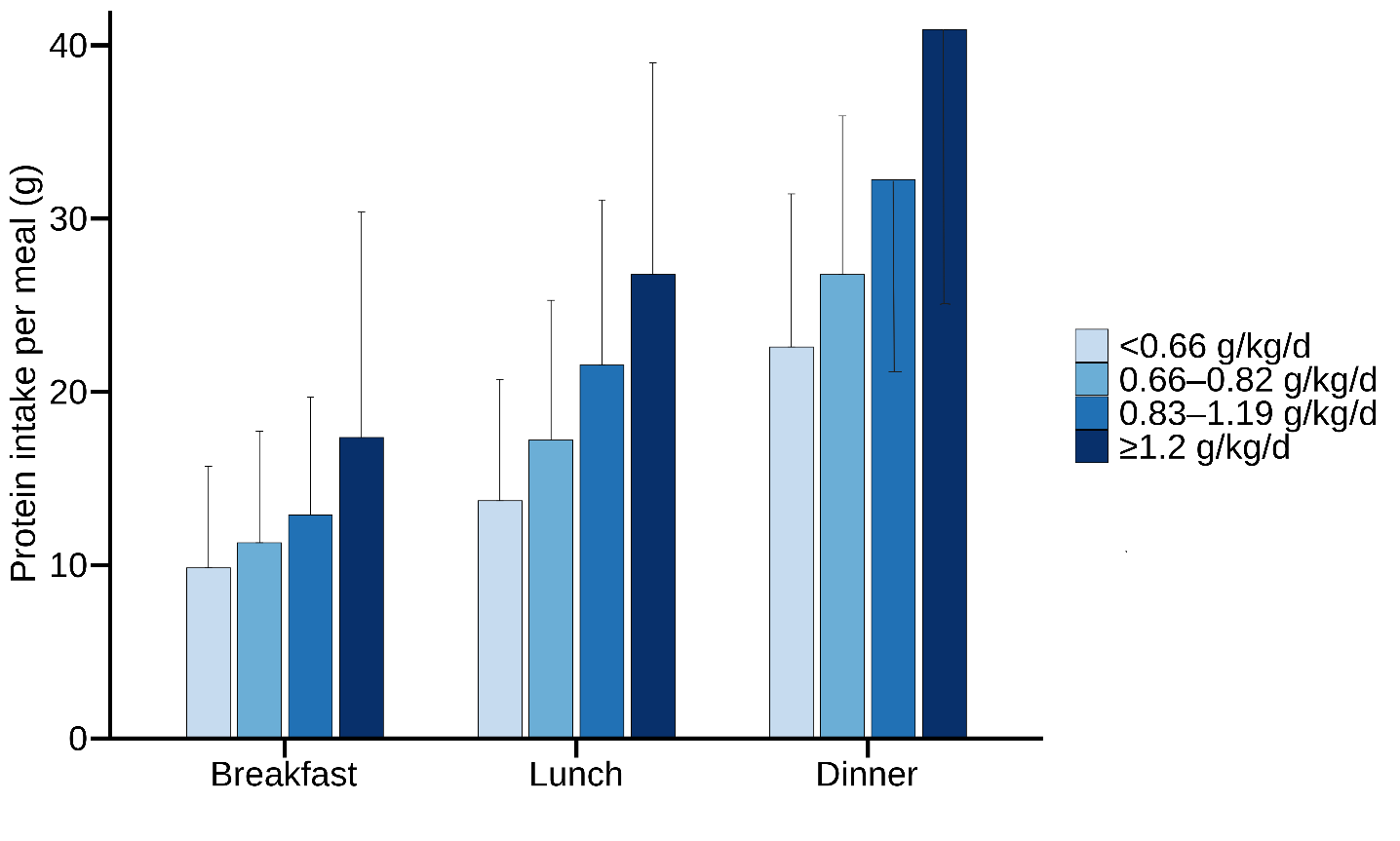
Supplementary figure 3** | Protein intake (g ± SD) per meal across different protein intake groups in participants consuming <60% plant-based protein
